# Stem cells purified from human induced pluripotent stem cell-derived neural crest-like cells promote peripheral nerve regeneration

**DOI:** 10.1038/s41598-018-27952-7

**Published:** 2018-07-03

**Authors:** Hiroo Kimura, Takehito Ouchi, Shinsuke Shibata, Tsuyoshi Amemiya, Narihito Nagoshi, Taneaki Nakagawa, Morio Matsumoto, Hideyuki Okano, Masaya Nakamura, Kazuki Sato

**Affiliations:** 1 Department of Orthopaedic Surgery, Keio University School of Medicine, 35 Shinanomachi Shinjuku-ku, Tokyo, Japan; 2 Department of Physiology, Keio University School of Medicine, 35 Shinanomachi Shinjuku-ku, Tokyo, Japan; 3 Department of Dentistry and Oral Surgery, Keio University School of Medicine, 35 Shinanomachi Shinjuku-ku, Tokyo, Japan; 4 Electron Microscope Laboratory, Keio University School of Medicine, 35 Shinanomachi Shinjuku-ku, Tokyo, Japan

## Abstract

Strategies for therapeutic cell transplantation have been assessed for use in the treatment of massive peripheral nerve defects. To support safe and efficient cell transplantation, we have focused on the purification of cells using cell surface markers. Our group previously reported low-affinity nerve growth factor receptor (LNGFR)- and thymocyte antigen-1 (THY-1)-positive neural crest-like cells (LT-NCLCs), generated from human induced pluripotent stem cells (hiPSCs). In the present study, we investigated the efficacy of transplantation of hiPSC-derived LT-NCLCs in a murine massive peripheral nerve defect model. Animals with a sciatic nerve defect were treated with a bridging silicone tube prefilled with LT-NCLCs or medium in the transplantation (TP) and negative control (NC) groups, respectively. The grafted LT-NCLCs survived and enhanced myelination and angiogenesis, as compared to the NC group. Behavioral analysis indicated that motor functional recovery in the TP group was superior to that in the NC group, and similar to that in the autograft (Auto) group. LT-NCLCs promoted axonal regrowth and remyelination by Schwann cells. Transplantation of LT-NCLCs is a promising approach for nerve regeneration treatment of massive peripheral nerve defects.

## Introduction

Tensionless nerve repair is an important advance in the surgical treatment of peripheral nerve injuries^1^. The current standard treatment for long-gap defects of peripheral nerves is autologous nerve transplantation^2^. However, harvesting autologous nerve grafts involves several challenges, such as donor-site morbidity, graft length limitation, and lengthy operation time^3–5^. Alternative approaches are needed to enable grafting of biomaterial devices into sites of injury. Artificial nerve conduits have been developed as one alternative^6–11^. Inside the conduit, an initial fibrin matrix that bridges the gap between nerve stumps is formed. The fibrin matrix provides a scaffold for the ingrowth of fibroblasts and blood vessels, and later of Schwann cells, which release multiple neurotrophic factors and lead to the axonal regrowth^12–15^. Although Schwann cells with artificial nerve conduits improve peripheral nerve regeneration^16–19^, the clinical use of such cells is limited by their source, purity, and immunologic rejection, and by potential ethical issues due to their autologous origin. In order to overcome these problems, various types of candidate cells, analogous to Schwann cells, have been tested.

We conducted the present study to identify better candidate donor cells for the treatment of massive peripheral nerve defects. We used human induced pluripotent stem cells (hiPSCs) as a cellular source in this study^20^. iPSCs are able to differentiate into various target cells under appropriate culture conditions. We induced neural crest-like cells from hiPSCs. Neural crest cells (NCCs) are known to derive from the ectoderm and can differentiate into neural lineage cells, including Schwann cells. Previous studies have reported the effectiveness of transplanting iPSC-derived NCCs for nerve regeneration^21–23^.

Mesenchymal stem cells (MSCs) have also been reported as a cell source for nerve regeneration^24–27^. MSCs are important players in tissue healing, and have been reported to exhibit the potential to differentiate into multiple cell types, including neural cells^28^. MSCs secrete various neurotrophic and angiogenic factors^24,29^. Many groups have attempted transplantation with MSCs into peripheral nerve injury models, with a view to achieving functional recovery^30–32^. Previous reports have indicated that NCCs share some of the same characteristics as MSCs^33^ and that some population of MSCs originate from NCCs during development^34,35^. We hypothesized that stem cells with the characteristics of both NCCs and MSCs might contribute to the functional recovery of massive peripheral nerve defects.

Mabuchi and colleagues reported that low-affinity nerve growth factor receptor (LNGFR) and thymocyte antigen-1 (THY-1) double-positive cells are a distinct MSC population in human bone marrow^36^. Previously, our group established a procedure for purifying a large number of LNGFR and THY-1 double-positive neural crest-like cells, designated as LT-NCLCs, from hiPSCs. The LT-NCLCs demonstrated a similar ability to NCCs and MSCs with regard to developing into Schwann-lineage cells^37^. Our group described the transplantation of LT-NCLCs within a silicone tube as a hybrid nerve conduit into a murine massive sciatic nerve defect. The purpose of the present study was to assess the efficacy of LT-NCLCs derived from hiPSCs for peripheral nerve regeneration and functional recovery.

## Methods

### iPSCs culture and NCLCs induction

The hiPSC lines WD39 and 201B7 were used in this study^20,38^. Human iPSCs were cultured in Matrigel-coated 6-well plates with mTeSR-1 (BD Bioscience, CA, USA). Medium was changed daily, and hiPSCs were passaged with collagenase IV (Thermo Fisher Scientific, MA, USA). LT-NCLC induction was slightly modified from that previously described^37^. hiPSCs were detached using collagenase IV and were then cultured in neural crest induction medium on 6-well adhesive dishes (Greiner Bio One, Kremsmünster Austria). Induction medium was composed of neurobasal medium (Thermo Fisher Scientific) and Dulbecco’s Modified Eagle’s Medium: Nutrient Mixture F-12 (Thermo Fisher Scientific) with 1% Gem 21 neuroplex (Gemini Bio-Products, CA, USA), 0.5% of x100 GlutaMax (Thermo Fisher Scientific), 0.5% N2 supplement (Thermo Fisher Scientific), 20 ng/ml of human epidermal growth factor (ReproTech, MO, USA), 20 ng/ml of human basic fibroblast growth factor (ReproTech), 20 ng/ml of insulin (Nacalai Tesque, Kyoto, Japan), and 0.5% penicillin and streptomycin. Induced cells formed spheres until day 4, and then formed spindle-shaped cells 9–10 days after induction.

### Flow cytometric analysis

The cell surface markers of the generated NCLCs were analyzed by flow cytometry. Dead NCLCs were eliminated by detection with propidium iodide (PI) (Sigma, MO, USA). Cells were using the antibodies described in the Supplementary Table 1. Cell surface markers of induced NCLCs were analyzed by using an Aria III flow cytometer (BD Bioscience). FlowJo software (Tree Star, OR, USA) was used for data analysis in this study.

### Immunocytochemistry of cultured cells

Sorted LNGFR- and THY-1-positive cells were cultured in neural crest induction medium in ultra-low attachment culture dishes for 1–2 days. Cells were aggregated, then transferred to 8-well plastic-bottomed glass chambers and cultured in the same medium for an additional 10 days. Cells were fixed by 4% paraformaldehyde (PFA) and washed with 0.1 M PBS, then blocked with blocking buffer (Nacalai Tesque) for 1 hour at room temperature. Cells were washed with 0.1 M PBS twice and applied the antibodies described in the Supplementary Table 1.

### Preparation of LT-NCLC-containing nerve conduits

Silicone tubes (NIHON EIDO, Co. Ltd., Tokyo, Japan; 1 mm i.d., 2 mm o.d.) used in this study were sterilized by autoclaving at 121 °C for 20 minutes. For tracking LT-NCLCs *in vivo*, cells were labeled with lentivirus for expressing *ff*Luc^39^, a green fluorescence protein (modified from Venus) fused to a luminescence protein (Luciferase 2) on days 3 and 9 under induction of NCLCs. LT-NCLCs, 2.0 × 10^5^ cells in medium were centrifuged at 800 g and the supernatant was discarded. Eighty microliters of the centrifuged LT-NCLCs were mixed with type I collagen (Cellmatrix I-A, Nitta Gelatin, Inc., Osaka, Japan) (40 µl) and reconstitution buffer (5 µl). The tube lumen was prefilled with LT-NCLCs containing collagen gel. The tube was cut into 8-mm sections, which were estimated to contain about 1.0 × 10^4^ cells each.

### Surgical procedures

To prepare the sciatic nerve injury model, immunodeficient NOD-SCID mice (6-week-old, males, Clea, Tokyo, Japan) were used. This experiment was approved by the Keio University Institutional Animal Care and Use Committee in accordance with the Institutional Guidelines (approval number: 17024-(0)).

All mice were deeply anesthetized with an intraperitoneal injection of ketamine (100 mg/kg; Sankyo, Tokyo, Japan) and xylazine (10 mg/kg; Bayer, Leverkusen, Germany). A dorsal longitudinal skin incision was made and the sciatic nerve was exposed by incising the gluteal fascia and bluntly splitting the gluteal muscle. The mice were assigned to four groups as described below. First, LT-NCLC-seeded collagen gel was injected into the silicone tube. The sciatic nerve was resected at the middle of the thigh. The gap was repaired by fixing the nerve stumps 1 mm inside the end of the tube using horizontal mattress suture of 9-0 monofilament nylon at each end, leaving a 6-mm interstump gap. Mice in the transplantation group (TP group, 16 mice) received a silicone tube filled with LT-NCLC-containing collagen gel. Mice in the negative control group received a silicone tube filled with acellular collagen gel to bridge the gap (NC group: 10 mice). As an Auto group, the 6-mm gap was reconstructed by turning the resected nerve over and bridging the resected nerve (Auto group: 10 mice). In the sham operation group, the sciatic nerves were simply explored without doing any damage to the nerve (Sham group: 3 mice). All procedures were carried out under a surgical microscope. As an *in vivo* analysis, survival of LT-NCLCs was continuously tracked. A luciferase substrate, D-luciferin (Summit Pharmaceuticals International Corporation, Tokyo, Japan) was subcutaneously injected into the animal (0.3 mg/g body weight) and the *ff*Luc-labeled cells were monitored using an *In Vivo* Imaging System (IVIS)-spectrum and CCD optical macroscopic imaging system (Caliper Life Sciences, MA, USA). Bioluminescence signals were measured every week after transplantation.

### Leg muscle contraction test

The motor function of the animals’ lower extremities was evaluated every two weeks using the leg muscle contraction test, in which isometric plantar flexion at the ankle is tested by pushing the sole until the toe touches the knee^40^. A digital force gauge (Nidec-Shimpo Corp., Kyoto, Japan) was used for measurement. The ratio of muscle contraction strength on the injured side compare to that on the contralateral side was averaged for each group.

### Walking Track Analysis

We also performed walking track analysis as another method of motor functional evaluation. Mice were placed on the treadmill instrument and their footprint was scanned using the DigiGait System (Mouse Specifics, MA, USA) at every two weeks after transplantation. We measured Sciatic functional index (SFI), which was calculated using the following fomulation^41^: SFI = 118.9 (ETS − NTS/NTS) − 51.2 (EPL − NPL/NPL) − 7.5 (ETS: experimental toe spread, NTS: normal toe spread, EPL: experimental print length, NPL: normal print length).

### Fluorescence Immunohistochemistry

Deeply anesthetized mice were used for histological analysis at 12 weeks after transplantation. Mice were perfused with 4% PFA in 0.1 M PBS, followed by removal of the sciatic nerve, and overnight fixation in 4% PFA in 0.1 M PBS, 10% sucrose, and then 30% sucrose. Silicone tubes were cut and removed immediately before fixation in 30% sucrose. Fixed sciatic nerves were embedded and frozen in Tissue-Tek optimal cutting temperature compound (Sakura Finetech Co., Ltd., Tokyo, Japan) and then sectioned in the axial and sagittal plane at a thickness of 16 µm on a CM3050 cryostat S (Leica Microsystems GmbH, Wetzlar, Germany). The primary and secondary antibodies described in the Supplementary Table 1 were applied. Sections were observed under an LSM780 and LSM880 confocal laser scanning microscope (Zeiss, Oberkochen, Germany).

### Electron microscopic analysis

Sciatic nerves from mice in the NC, TP, Auto, and Sham group (n = 3 from each group) were used for electron microscopic (EM) observation, as described previously^42^. Briefly, the tissues were dissected and fixed with 2.5% glutaraldehyde in 0.1 M phosphate buffer (PB) (pH 7.4) for 24 hours at 4 °C. After two hours of post-fixation with 1% OsO_4_, tissues were dehydrated through a graded concentration of ethanol, acetone, with n-butyl glycidyl ether (QY1), exposed to a graded concentration of Epon with QY-1, and finally to 100% Epon for 72 hours to enhance the infiltration. Pure Epon embedded tissues were polymerized for 72 hours at 60 °C. Semi-thin sections with 1-μm thickness were stained with 0.1% toluidine blue for 7 minutes, and imaged with BZ9000 (Keyence, Osaka, Japan). Ultrathin sections (70-nm thickness) of the axial sciatic nerve were prepared with an ultramicrotome (Leica UC7, Leica Microsystems GmbH) on copper grids and silicon wafer, and stained with uranyl acetate and lead citrate for 10 minutes. The sections were observed under a transmission electron microscope (JEM-1400Plus, JEOL Ltd., Tokyo, Japan), and scanning electron microscope (multiSEM505, Zeiss). For quantitative G-Ratio analysis, the diameters of the myelinated nerve fibers in TP group (total 109 fibers) and Auto group (total 142 fibers) were evaluated from randomized EM images (n = 3 mice from each group).

### Immuno-electron microscopy (iEM)

Frozen sections of sciatic nerve were used for iEM analysis with previously described procedure^42^. Briefly, the 20-μm-thick frozen sections were incubated with 5% block ace (DS Pharma Biomedical, Osaka, Japan) with 0.01% saponin in 0.1 M PB for an hour. Sections were stained with primary mouse STEM121 antibody (1:500) for 72 hours at 4 °C, followed by incubation with nanogold-conjugated goat anti-mouse secondary antibody (1:100 Thermo Fisher Scientific) for 24 hours at 4 °C. After 2.5% glutaraldehyde fixation, nanogold signals were enhanced with R-Gent SE-EM Silver Enhancement Reagents (Aurion, Wageningen, Netherlands) for 30 minutes. Sections were post-fixed with 1.0% OsO_4_ for 90 minutes at 25 °C, dehydrated through a graded series of ethanol and embedded into 100% Epon. The ultrathin sections with electron staining were observed under a transmission electron microscope.

### Statistical analysis

All recoded data are presented as the mean ± the standard error of the mean (SEM). For histological examinations, the Kruskal-Wallis test followed by the Steel test was used for multiple comparisons among the four groups. For behavior analysis, Welch’s t-test was performed between the TP and NC group, and the TP and Auto group. JMP 12 (SAS Institute, NC, USA) was used for all statistical analyses.

## Results

### Induction of NCLCs and sorting of the LT-NCLCs from hiPSCs

**Figure 1:**
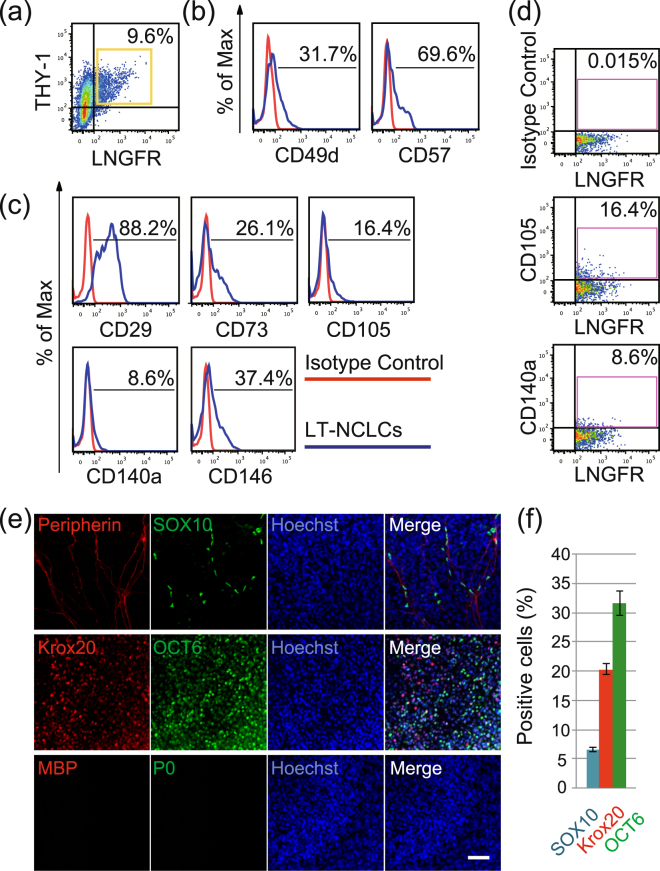
Characterization of LT-NCLCs by flow cytometry. (**a**) Neural crest-like cells (NCLCs) derived from human induced pluripotent stem cells (hiPSCs) line (WD39) partially expressed CD271 (low-affinity nerve growth factor receptor; LNGFR) and CD90 (thymocyte antigen-1; THY-1). (**b**–**d**) Flow cytometric analysis revealed that the LNGFR- and THY-1-positive neural crest-like cells (LT-NCLCs) expressed neural crest cells (NCCs) markers (CD49d and CD57) and mesenchymal stem cells (MSCs) markers (CD29, CD73, CD105, CD140a, and CD146 respectively). (**e**) The LT-NCLCs maintained in NCC medium expressed the neural crest lineage marker, SOX10, and the neural markers, Peripherin (upper panels), Krox20, and OCT6 (middle panels), but did not express the myelinating cell markers P0 and MBP (lower panels). Scale Bar: 50 µm. (**f**) Quantitative analyses for SOX10, Krox20, and OCT6-positive cells were performed in Hoechst-positive cells.

To prepare the cell source, we generated NCLCs from the hiPSC line WD39. Induced cells formed spheroids by day 4 after induction, and then spindle-shaped cells migrated from spheroids under adherent culture conditions. Flow cytometric analysis revealed that 9.6 ± 0.3% LT-NCLCs were generated (Fig. 1a). LT-NCLCs were generated from 201B7, another iPSC line, and evaluated by flow cytometry. The induction efficiency of LT-NCLCs from 201B7 for LNGFR(+)THY-1(+) was 20.5 ± 0.8%, suggesting the reproducibility of NCLC induction using another iPSCs line (Supplementary Fig. 1).

The LT-NCLCs, LNGFR and THY-1 double-positive fraction, partially expressed neural crest markers CD49d (31.7 ± 1.5%), CD57 (69.6 ± 1.1%) (n = 4) (Fig. 1b). The LT-NCLCs also expressed MSCs markers CD29 (88.2 ± 2.3%), CD73 (26.1 ± 1.3%), CD105 (16.4 ± 1.2%), CD140a (8.6 ± 0.5%) and CD146 (37.4 ± 2.6%) (n = 4) (Fig. 1c,d). These data indicated that LT-NCLCs expressed both MSCs and NCCs markers. To evaluate the LNGFR/THY-1 single-positive [LNGFR(+)THY-1(−), LNGFR(−)THY-1(+)], and double-negative [LNGFR(−)THY-1(−) cells] fractions, we performed flow cytometric analysis with the NCC/MSC markers, including CD49d, CD57, CD29, CD73, CD105, CD140a and CD146 (Supplementary Fig. 2). Expression level of NCC markers (CD49d and CD57) and MSC markers (CD29, CD73, CD105, CD140a, and CD146) in these LNGFR/THY-1 single-positive and double-negative fractions were described in Table 1 and Supplementary Fig. 2a–c. These results demonstrated that the single positive fraction and double negative fraction showed the lower levels of expression for each NCC/MSC markers.

**Table 1:** Summary of neural crest and mesenchymal lineage marker expression.

Fraction	NCC	NCC	MSC	MSC	MSC	MSC	MSC
	CD49d	CD57	CD29	CD73	CD105	CD140a	CD146
LNGFR(+)THY-1(+)	31.7 ± 1.5%	69.6 ± 1.1%	88.2 ± 2.3%	26.1 ± 1.3%	16.4 ± 1.2%	8.6 ± 0.5%	37.4 ± 2.6%
LNGFR(+)THY-1(−)	3.3 ± 2.4%	60.5 ± 6.0%	44.4 ± 7.1%	0.4 ± 0.4%	0.2 ± 0.2%	0.0 ± 0.0%	2.5 ± 2.5%
LNGFR(−)THY-1(+)	10.1 ± 1.2%	40.3 ± 1.5%	62.1 ± 4.5%	9.6 ± 0.6%	5.7 ± 1.2%	1.7 ± 0.2%	15.3 ± 1.6%
LNGFR(−)THY-1(−)	0.8 ± 0.2%	40.8 ± 3.4%	14.0 ± 1.1%	0.4 ± 0.1%	0.4 ± 0.1%	0.1 ± 0.0%	0.7 ± 0.2%

The proportion of the NCC markers (CD49d and CD57) and MSC markers (CD29, CD73, CD105, CD140a, and CD146) in the LNGFR/THY-1 double positive, single-positive and double-negative fractions were evaluated by FACS analysis (related to Fig. 1 and Supplementary Figure 2). The expression level for NCC/MSC markers was the highest in LNGFR(+)THY-1(+) fraction compared to that in other single positive and double negative fraction.

There is a possibility that the LT-NCLCs consisted of some NCC/MSC marker double-positive cell population or from a mixed population of cells single-positive for NCC/MSC markers. To address this, flow cytometric analysis was performed to evaluate the existence of NCC/MSC marker double-positive cells with NCLC induction. From 1.0 to 11.2% of the cells were double-positive fraction for NCC/MSC markers, combination with the antibodies for LNGFR (CD271), CD49d as NCC markers and CD29, CD73, CD105, CD140a, CD146 and THY-1 (CD90) as MSC markers (Supplementary Fig. 3). In addition, LT-NCLCs in NCC medium were immunostained with the NCC marker CD57 and the MSC marker CD140a (Supplementary Fig. 4). Some differentiated LT-NCLCs were double-positive for both NCC/MSC markers; others were single-positive for NCC/MSC markers. We also investigated the expression profiles of core transcriptional factors related to NCC and MSC development and differentiation^43,44^, including FoxD3, Slug, AP2α, SOX9, Pax3, Pax7, FoxP1, GATA6, Runx2, and PPARγ (Supplementary Fig. 5a). The results of immunocytochemistry show that differentiated LT-NCLCs express multiple transcriptional factors involved in NCC and MSC lineage differentiation. Quantitative analyses of these images were performed using the software in BZ9000, using antibody staining against these transcriptional factors counterstained with Hoechst. The frequencies of positive cells among the total LT-NCLCs were as follows; FoxD3; 91.2 ± 1.2%, Slug; 86.1 ± 1.7%, AP2α; 82.0 ± 1.2%, SOX9; 77.2 ± 1.6%, Pax3; 50.4 ± 1.7%, Pax7; 37.1 ± 2.0%, FoxP1; 39.6 ± 3.6%, GATA6; 29.5 ± 3.1%, Runx2; 0.4 ± 0.2%, and PPARγ; 0.2 ± 0.1% (Supplementary Fig. 5b). These results suggest that differentiated LT-NCLCs show slightly stronger NCC phenotypes than MSC phenotypes.

### Characterization of the LT-NCLCs *in vitro*

To investigate the characteristics of LT-NCLCs, immunocytochemistry was performed. Multicolor immunofluorescence staining of LT-NCLC-derived cells cultured in neural crest medium showed that SOX10-positive cells were in the vicinity of the Peripherin-positive area (Fig. 1e upper panels). SOX10 is a marker of neural crest stem/progenitors and Schwann lineage cells. The LT-NCLC-derived cells were also positive for the neural markers Krox20 and OCT6, which are markers of immature Schwann cells (Fig. 1e middle panels). However, they were not positive for the myelinating mature Schwann cell markers, MBP and P0 (Fig. 1e lower panels). These findings indicate that the neural crest stem/progenitors or immature Schwann cells were developed by neural crest induction. Quantitative analyses were carried out with the multiple images of immunocytochemistry stained with anti-human SOX10, Krox20, and OCT6, counterstained with Hoechst. SOX10, Krox20, and OCT6-positive cells accounted for 6.6 ± 0.4%, 20.3 ± 0.9%, and 31.6 ± 2.1% of the total cells, respectively (Fig. 1f).

### Survival, proliferation, and migration of transplanted LT-NCLCs

**Figure 2:**
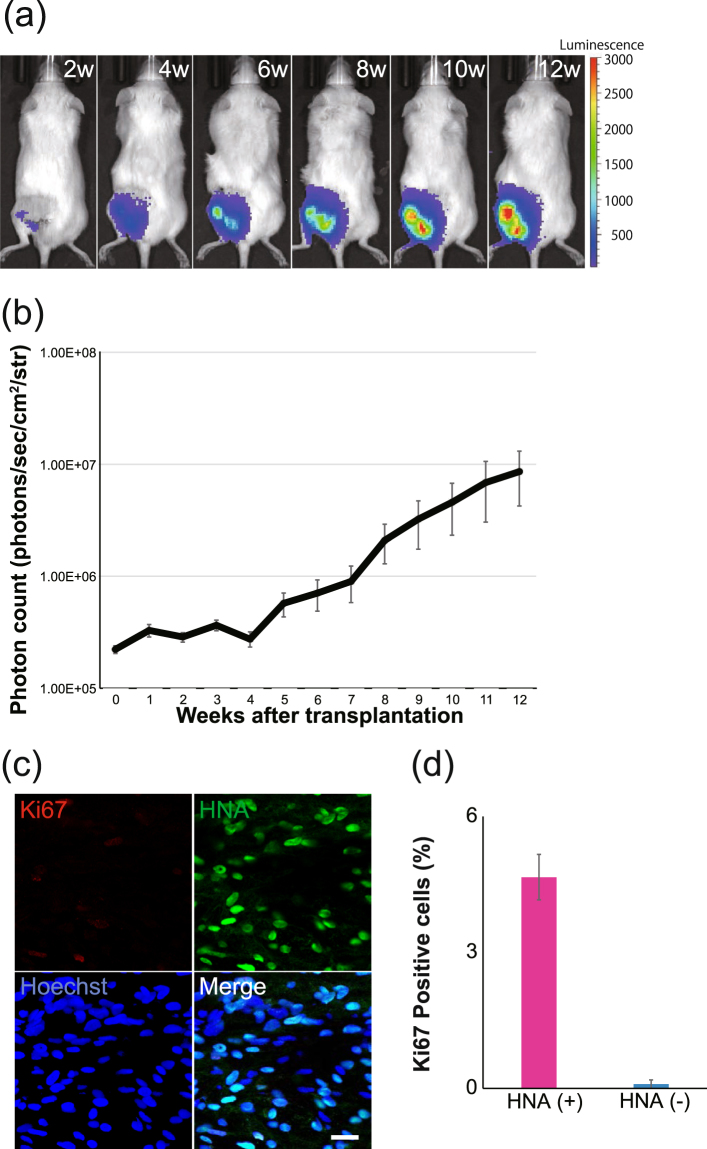
Bioluminescence tracking of transplanted cells. (**a**) Representative bioluminescence images of the mouse at every two weeks after transplantation. Luminescence increased until 12 weeks after transplantation. (**b**) Quantitative analyses of the photon count derived from transplanted cells until 12 weeks. The *in vivo* survival and proliferation of transplanted cells were observed. (**c**) Fluorescence images of immunohistochemistry in the TP group performed with staining for HNA, Ki67, and Hoechst in sagittal sections at 12 weeks after transplantation. Scale bar, 50 µm. (**d**) Ki67-positive cells in HNA-positive and -negative cells were quantitatively analyzed. Most of the Ki67-positive cells were derived from human engrafted LT-NCLCs.

To examine the efficacy of LT-NCLCs *in vivo*, we transplanted the cells in the injured sciatic nerve of NOD-SCID mice (TP group) and observed its effects over time. Surgical procedures were also performed in the NC, Auto, and Sham groups (Supplementary Fig. 6). Cell tracing in *in vivo* imaging system (IVIS) showed that grafted LT-NCLCs survived for 12 weeks (Fig. 2a). Luminescence photon counts increased gradually, which suggested that LT-NCLCs proliferated after transplantation (Fig. 2b). To investigate the survival and proliferative potential of transplanted cells inside the conduit, sections were stained with an anti-HNA antibody and an anti-Ki67 antibody against the cell cycle regulation protein (Fig. 2c). In the center portion of the regenerated nerves in the TP group, 70.2 ± 3.5% of cells were HNA-positive, which originated from LT-NCLCs. The Ki67-positive cells accounted for 4.7 ± 0.5% of HNA-positive cells. Quantitative analysis of the dividing cells was performed at the center potion of regenerated nerve fibers in TP group (Fig. 2d). Most of the Ki67-positive proliferating cells were positive for HNA, suggesting that some engrafted LT-NCLCs showed a certain level of proliferative activity at 12 weeks after transplantation.

The migration activity of the grafted cells was evaluated the HNA-positive cells on the sagittal section of the transplanted sciatic nerve with immunohistochemistry (Supplementary Fig. 7). HNA-positive cells were evaluated in proximal, center, and distal portion of the regenerated nerve fiber. There were no significant differences of the HNA-positive cell distribution among these three portions. This suggests that the grafted cells actively migrated and became equally distributed throughout the nerve conduit in the silicone tube.

### Leg motor functional recovery via LT-NCLC transplantation

**Figure 3:**
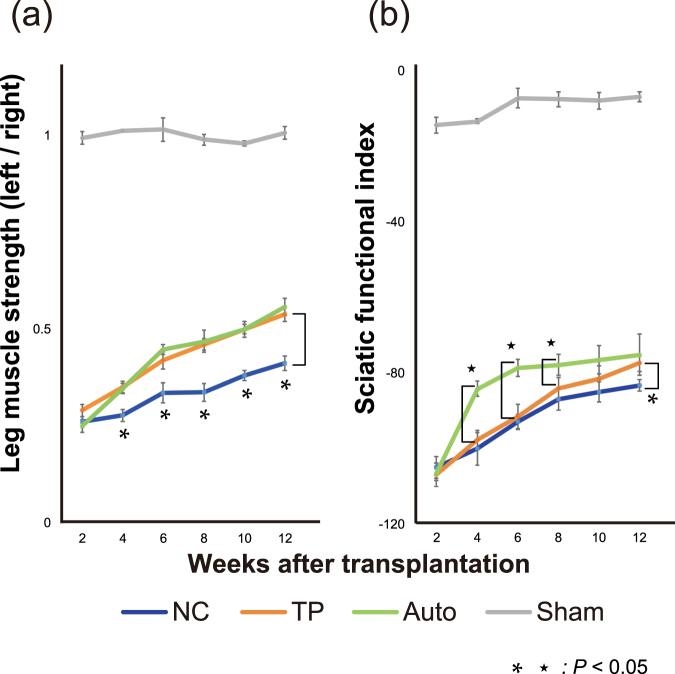
Functional recovery promoted by cell transplantation. (**a**) Leg muscle contraction tests showed that functional recovery was significantly better in the transplantation (TP) group than in the negative control (NC) group on and after four weeks after transplantation. (b) Walking track analysis revealed that the sciatic functional index in the TP group was higher than in the NC group at 12 weeks after transplantation. Both analyses demonstrated that motor functional recovery in the TP group was eventually equal to that in the autograft (Auto) group at 12 weeks after transplantation.

Leg muscle contraction tests were conducted to assess the strength of the muscle innervated by the sciatic nerve. The leg muscle strength in the TP group was significantly higher than that in the NC group at 4–12 weeks after transplantation (Fig. 3a). There was no significant difference between the TP group and the Auto group. Walking tracks were also measured to calculate SFI, which indicate the functional recovery of the sciatic nerve. The SFI in the TP group was also significantly higher than that in the NC group at 12 weeks after transplantation (Fig. 3b). The SFI in the Auto group was significantly superior to that in the TP group in the early period, but there were no significant differences in the later period. These results indicated that transplantation of LT-NCLCs promoted motor functional recovery of the sciatic nerve equal to that promoted by autograft transplantation.

### Promoted regeneration of nerve fibers by LT-NCLC transplantation

**Figure 4:**
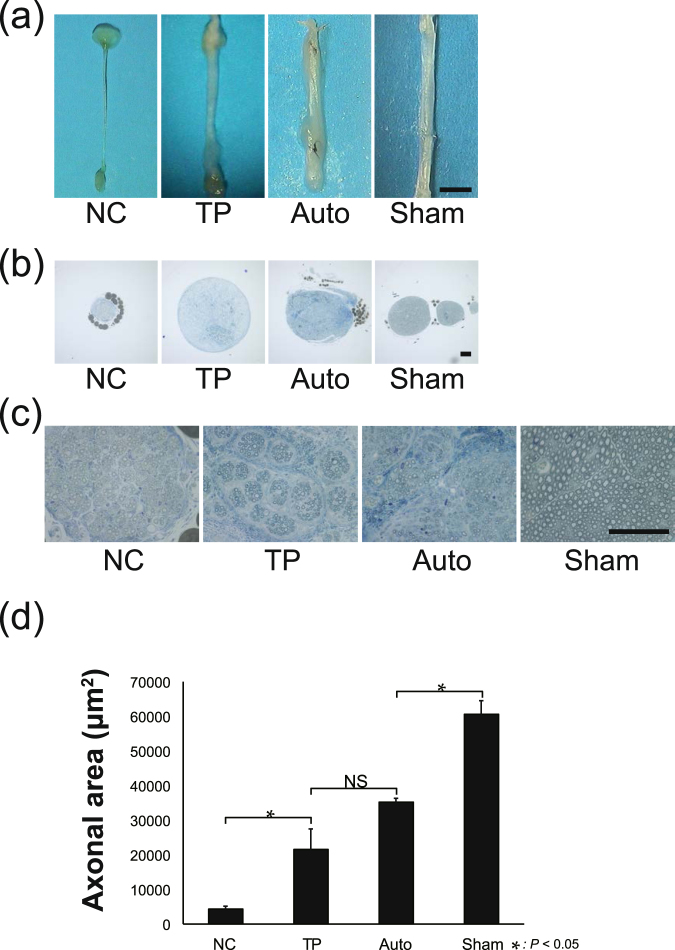
Enhanced nerve regeneration in the TP group. (**a**) Representative images of a sciatic nerve at 12 weeks after transplantation. Thick regenerated nerve fibers were observed in the TP group. (**b**,**c**) Toluidine blue staining in middle axial sections revealed that the regenerated nerve fiber in the TP group contained numerous axons. Scale bar, 100 µm. (**d**) Quantitative analysis of the axonal area of regenerated nerve fibers (axonal area = cross-sectional area × axon density). There were significant differences between the TP and NC groups, and also between the TP and Sham groups (*P* < 0.05). NS, non-significant.

In order to evaluate the histological recovery *in vivo* 12 weeks after transplantation, the sciatic nerves from each group were fixed and observed under an optical microscope. Previous studies have shown that peripheral nerve regeneration using conventional nerve conduits usually fails with gaps 6 mm or longer in mice^6–8^. In the TP group, all 16 specimens showed nerve regeneration inside the conduits. In the NC group, seven of 10 specimens had regenerated nerve bridges, but the remaining three specimens showed empty conduits. A thick regenerated nerve fiber was observed in the TP group (Fig. 4a). The toluidine blue-staining images of axial sections demonstrated that the diameter of regenerated nerve fibers was quite large in the TP group (Fig. 4b) and a substantial number of myelinated axons were observed inside the regenerated nerves (Fig. 4c). Quantitative analysis of axonal areas showed that the TP group had a significantly higher count than the NC group, and this analysis indicated that LT-NCLC transplantation promoted axonal regrowth (Fig. 4d).

### Transplanted LT-NCLCs enhance host myelination

**Figure 5:**
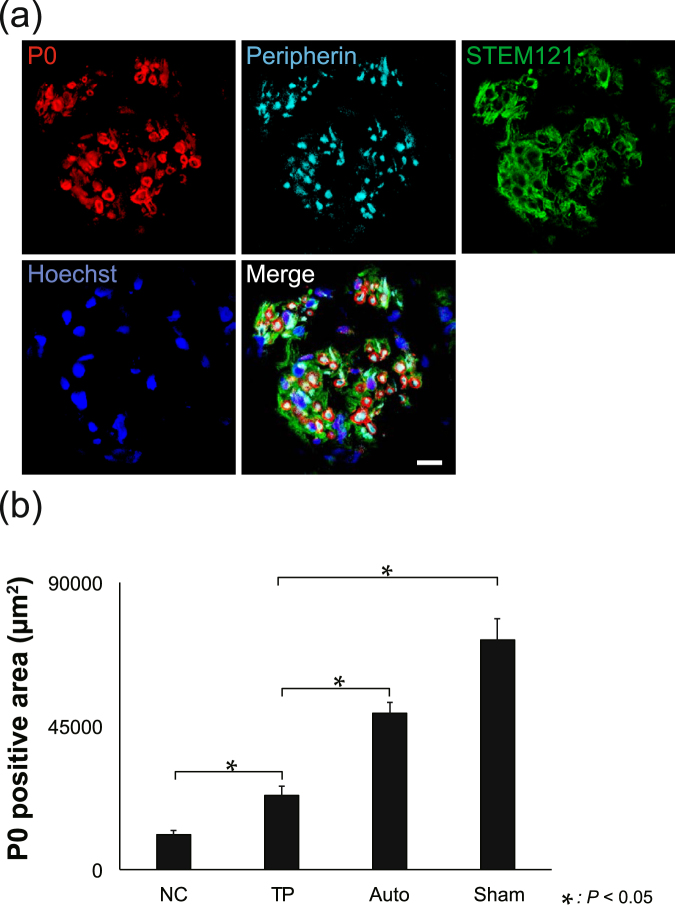
P0-positive mature myelination in the TP group. (**a**) Representative images of immunohistochemistry stained with anti-human cytoplasm (STEM121), anti-P0, and anti-Peripherin antibodies, counterstained with Hoechst, in middle axial sections at 12 weeks after transplantation. STEM121-staining was observed at the margin of the ring-shaped P0-positive region. The center of this region showed staining for Peripherin. These findings showed that transplanted cells enhanced regeneration of matured Schwann cells and myelinated axons. Scale bar, 10 µm. (**b**) Quantitative analysis of the P0-positive area of axial sections. There were significant differences between the TP and NC groups (*P* < 0.05).

To assess the distribution and differentiation of transplanted cells, we performed immunostaining of sections 12 weeks after transplantation (Fig. 5a). Immunostaining assays were also performed in the NC, Auto, and Sham groups (Supplementary Fig. 8). The P0-positive area was adjacent to the human cytoplasm-positive area, which labeled with the STEM121 antibody. Quantitative analysis of the P0-positive area was carried out. P0-positive area of the TP group was significantly larger than that of the NC group, which indicated that host myelination was promoted by transplanted cells (Fig. 5b).

### Active myelination and angiogenesis under electron microscopic observation

**Figure 6:**
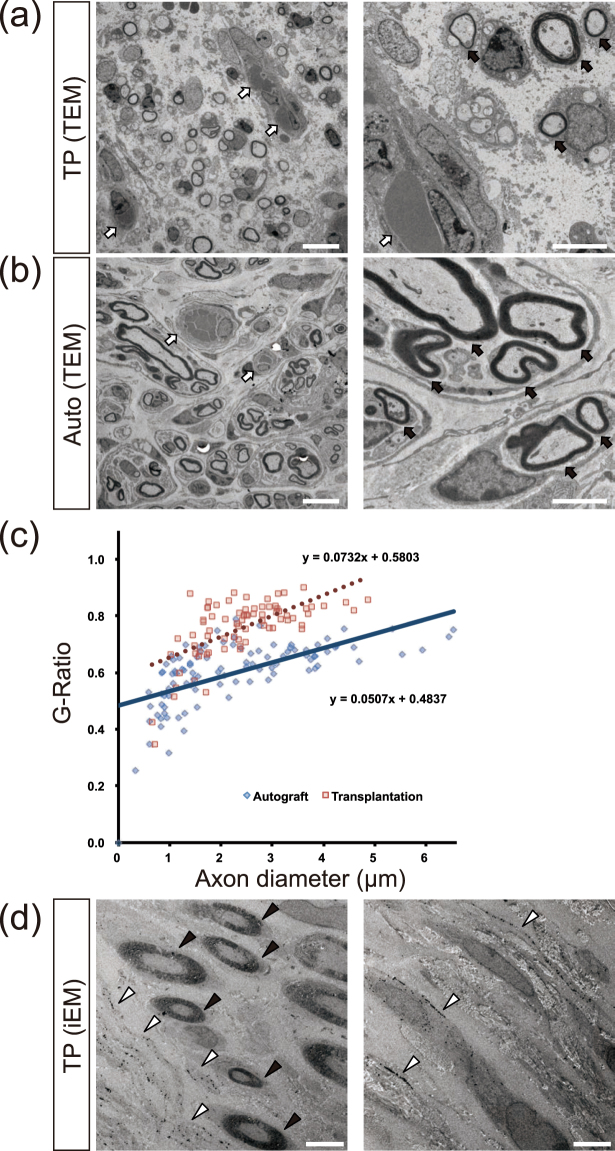
Active myelination and vascularization detected by electron microscopy. (**a**) Representative electron microscopic images in central axial sections of the TP group showed that a large number of regenerated myelinated axons (black arrows) as well as vascular formation (white arrows) were detected. Scale bars, 10 µm (left), 5 µm (right). (**b**) Transmission electron microscopic (TEM) images of the Auto group showed that regenerated myelinated axons were detected (black arrows), similar to in the TP group. Vascular formation was also detected (white arrows). Scale bars, 10 µm (left), 5 µm (right). (**c**) Quantitative analysis of myelination from the TP and Auto groups was carried out. G-Ratio of each group indicated that the thickness of the myelin lamellae of TP group was thinner than that of the Auto group. Red square dots: TP group, blue diamond dots: Auto group. (**d**) Representative images of immunoelectron microscopy (iEM) revealed that transplanted cells were detectable by the black dots observed upon STEM121 antibody staining (white arrow heads). STEM121 antibody labeling was observed in the cytoplasm of the cells surrounding myelin sheaths and also in the cytoplasm of myelinated Schwann cells (black arrow heads). Scale bars, 10 µm (left), 5 µm (right).

In order to identify the detailed histological recovery *in vivo*, electron microscopic analysis was carried out on the sciatic nerve at 12 weeks after surgery. A large number of regenerated axons myelinated by Schwann cells was observed in axial sections of the transplanted sciatic nerve (Fig. 6a) in the TP group, similar to the Auto group (Fig. 6b). Active vascular formation with erythrocytes surrounded by endothelial cells was also detected in the sciatic nerve in the TP group. Quantitative evaluation of the myelination from TP and Auto groups was carried out to determine the G-Ratio for each group (Fig. 6c). The average data of the axonal diameter was TP (2.37 ± 0.97 µm), Auto (2.51 ± 1.47 µm) and that of G-Ratio was TP (0.76 ± 0.11 µm), Auto (0.62 ± 0.083 µm). These results indicate that the thickness of the myelin lamellae of TP group was thinner than that of the Auto group.

To distinguish the transplanted human cells from endogenous mouse cells under electron microscopy, iEM analysis was carried out with frozen sections of the sciatic nerve by using the anti-human cytoplasm STEM121 antibody to detect human cytoplasm (Fig. 6d). The gold-labeled STEM121 positive dots were localized adjacent to the myelin sheaths (Fig. 6d left). Most of the myelinating cells’ cytoplasm was not labeled with the STEM121 antibody (Fig. 6d left). The black positive dots were frequently localized between the collagen fibers arranged in a matrix pattern in ultrathin sections of the regenerating sciatic nerve (Fig. 6c right). iEM observation clearly indicated that many transplanted human cells survived in the regenerating sciatic nerve, and they actively enhanced host cell myelination.

### Active angiogenesis is enhanced by LT-NCLC transplantation

**Figure 7:**
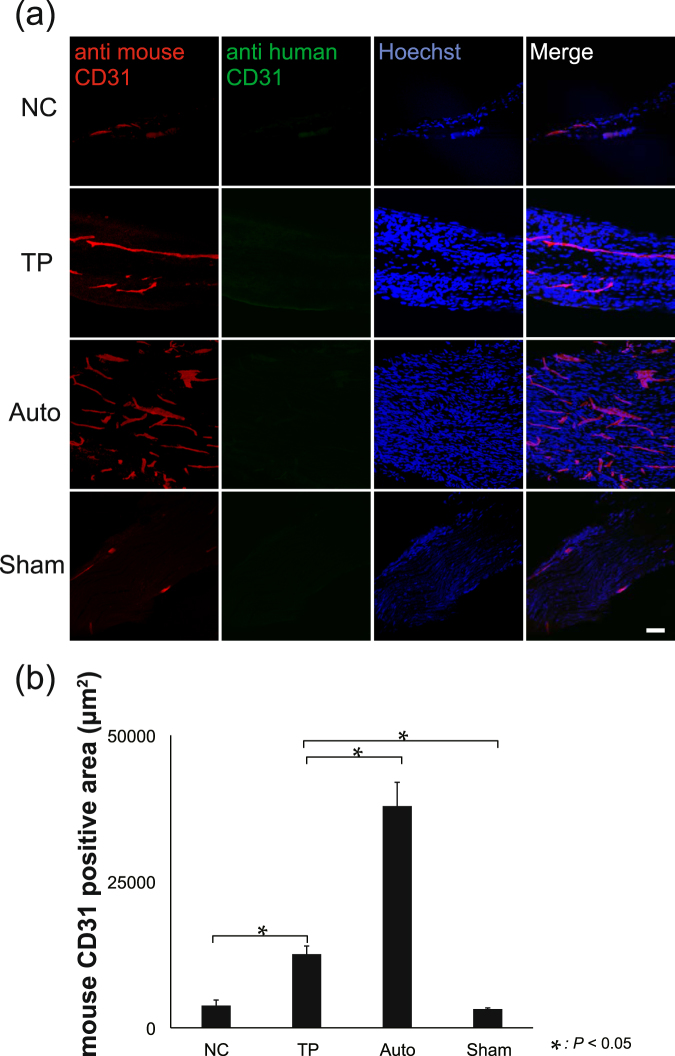
CD31-positive endothelial cells of murine origin. (**a**) Immunohistochemical staining for anti-human CD31, anti-mouse CD31, and Hoechst in the four groups demonstrated that host endothelial cells were recruited into the conduit; however, transplanted cells did not differentiate into endothelial cells. Scale bar, 50 µm. (**b**) Quantitative analysis of the anti-mouse CD31-positive area in sciatic nerve. There were significant differences between the TP and NC groups (*P* < 0.05).

To evaluate the origin of angiogenesis inside the conduits of the TP group, immunostaining with species-specific anti-CD31 antibodies was performed (Fig. 7a). The vascular endothelial cells at newly formed blood vessels stained positive with anti-mouse CD31 antibody, but negative with anti-human CD31 antibody. The mouse CD31-positive area was quantitatively analyzed (Fig. 7b). There were significant differences among groups, suggesting that LT-NCLCs actively recruited host cell-derived angiogenesis. In this trial, LT-NCLCs showed limited potential for differentiation into endothelial cells.

### Mechanisms for the nerve regeneration and angiogenesis by LT-NCLC transplantation

Next, to determine the mechanisms for enhanced regeneration of nerve fibers and angiogenesis *in vivo*, we evaluated the NCC/MSC markers in grafted cells inside the conduits of the TP group. This was detected by immunostaining with NCC marker CD49d and MSC marker CD146 that these NCC/MSC markers were disappeared at 12 weeks after transplantation (Supplementary Fig. 9). This result suggests that active functional recovery was not mainly attributable to the surviving NCC/MSC marker-positive cells, but rather to the descendants of differentiated LT-NCLCs *in vivo*.

Finally, to confirm the expression profiles of the neurotrophic and angiogenic factors in LT-NCLCs, immunohistochemistry was performed with the differentiated LT-NCLCs and iPSCs *in vitro* (Supplementary Fig. 10). Differentiated LT-NCLCs began to express BDNF and VEGF compared to the iPSCs, suggesting that the active expression of neurotrophic factors and angiogenic factors supports the enhanced recovery of the peripheral nerve regeneration.

## Discussion

In this study, transplanted LT-NCLCs promoted host axonal regrowth, remyelination of Schwann cells, and recruited angiogenesis inside conduits (Figs 4–7). Grafted LT-NCLCs showed strong survival and finally contributed to the improvement of motor functional recovery (Figs 2 and 3). Numerous stem cell transplantation strategies have been attempted for nerve regeneration^21–27,30–32^; there is a limited chance that unknown dangerous cells may contaminate the preparation without applying purification. Transplantation of a heterogeneous cell population creates ambiguity about which type of cells critically contributed to nerve regeneration. We demonstrated the contribution of LT-NCLCs by purifying them by using two cell surface markers, LNGFR and THY-1, using flow cytometry. It was reported that LT-NCLCs are composed of NCC and MSC lineage cells^37^; in fact, LT-NCLCs express NCC and MSC markers, including CD49d and CD57 as evaluated by flow cytometry (Fig. 1). Differentiated LT-NCLCs expressed the neural markers, SOX10, Peripherin, OCT6 and Krox20. It is quite reasonable to explain the role of LT-NCLCs due to the cell purification steps and the rigidly controlled induction steps.

Previously, NCCs and their descendants, including Schwann cells, have been widely used in interventions for peripheral nerve damage. Several studies have demonstrated that transplanted Schwann cells promoted axonal regeneration^16–19^. In addition, some studies reported that combination of Schwann cells and other types of cells transplantation, such as neurons^45^, stem cells^46^, and nerve fibroblasts^47^, promoted nerve recovery. Variety of past studies showed the therapeutic efficacy of NCCs transplantation^21,22^. Okawa *et al*. demonstrated that grafted NCLCs derived from mouse iPSCs improved murine diabetic neuropathy^22^. According to this report, grafted cells not only secreted various kinds of trophic factors, but also differentiated into smooth muscle cells or Schwann-like cells to contribute the recovery. The optimal cell type for transplantation—NCCs and/or differentiated Schwann cells—remains controversial. To date, Huang *et al*. evaluated whether the differentiation stage of transplanted stem cells modulates nerve regeneration^23^. They compared the transplantation performance of iPSC-derived neural crest stem cells (NCSCs) and Schwann cells from the NCSCs (NCSC-SCs) in rat sciatic nerve transection models. Accelerated motor functional recovery was supported by a larger amount of BDNF and nerve growth factor (NGF) secreted from transplanted NCSCs, compare to those from NCSC-SCs. They mention that the combination of the differentiation stage and paracrine signaling of the transplanted cells were strongly related to the efficiency for the cell transplantation therapy.

About MSCs, various studies demonstrated a lot of promising effects of MSCs transplantation. As well as infusion of trophic factors, transplantation of MSCs also has several advantages for nerve regeneration. It was reported that bone marrow-derived MSCs can differentiate into a cell with Schwann cell-phenotype and support neurite growth and axonal regeneration, quite similar to Schwann cells^26^. Wang J *et al*. also demonstrated that bone marrow-derived MSCs co-cultured with Schwann cells not only enhanced cell survival and proliferation of Schwann cells, but also stimulated the expression of NGF, BDNF, TrkA and LNGFR on Schwann cells *in vitro*, and enhanced Schwann cell proliferation and axonal outgrowth *in vivo*^27^. Recently, it has been reported that MSCs escape recognition by the immune system by inhibiting the immune response^48^, which is a favorable property for the survival of grafted cells survival *in vivo*. Additionally, Cooney *et al*. reported that locally injected MSCs against rat sciatic nerve injury could modulate inflammatory conditions and reduced fibrosis of the regenerated nerve, which resulted in increased the count of nerve fiber and functional recovery^30^. In our study, we speculated that these immunologic features of MSCs might deliver some beneficial effect for the grafted cell survival and proliferation. Contrary to these indirect effects from transplanted MSCs, the direct contribution from transplanted MSCs was also documented by Dezawa *et al*.^25^. That study showed that differentiated MSCs begin to express Schwann cell markers by themselves, and may be capable of forming myelin and directly facilitated sciatic nerve regeneration. In our study, there were a variety of benefits of LT-NCLC transplantation, including trophic factor expression, recruitment of blood vessels, and remyelination, for enhanced functional recovery after nerve defects.

Motor functional assessment in this study showed that the TP group was significantly superior to the NC group and almost equal to the Auto group (Fig. 3). Toluidine blue staining revealed that axonal regrowth was strongly promoted by LT-NCLC transplantation (Fig. 4), suggesting a strong correlation between axonal area and motor functional recovery. For good functional recovery, both axonal regrowth from the host neuron and remyelination is crucial and was supported by the transplanted cells. Some previous reports have demonstrated that most of the transplanted cells directly contributed to the recovery, by themselves^22,25^. In our study, remyelination and revascularization were present at the injury site; however, most of the myelinating Schwann cells and endothelial cells were derived from the host cells (Figs 5–7), supported by the LT-NCLC transplantation. As for the neurotrophic and angiogenic factor expression, our differentiated LT-NCLCs express BDNF and VEGF on *in vitro* analysis (Supplementary Fig. 10). The effect of BDNF and VEGF in peripheral nerve injuries has been shown previously^49,50^; these factors may play important roles in introducing host blood vessels and Schwann cells during peripheral nerve recovery. These indirect contributions to recovery suggested that the LT-NCLCs create an optimal niche for peripheral nerve regeneration.

For future clinical applications, the donor cell source is important for both recovery and safety. hiPSCs were first established in 2007^20^. hiPSCs have a great advantage as a cell source thanks to the establishment of an iPSC bank suitable for HLA-matched cell transplantation^51,52^. hiPSCs also have the advantage of offering a limitless supply of donor cells due to their active proliferation potential. There is a concern about the possibility of tumorigenic transformation when it comes to the clinical use of stem cells, especially hiPSCs. To date, no tumor formation has been detected in our study; however, the photon counts of bioluminescence images gradually increased until 12 weeks after transplantation and a certain number of Ki67-positive human cells were detected. The results indicated that the grafted cells were supposed to maintain a proliferative potential at 12 weeks after transplantation. Ki67 is an immunocytochemical marker of cell proliferation and, is known clinically as a prognostic biomarker for variety of tumors, especially for breast cancer^53,54^ and for neuroendocrine tumors (NETs)^55^. Past studies have reported a linear correlation between the Ki67-positive rate of the grafted stem cells and tumor formation^56,57^. Clinically, there are several cut-off lines of the Ki67-positive rate for cancers. For breast cancer diagnosis, several groups demonstrated the Ki67-positive rate and prognosis assessment. One cohort study analyzed by Cheang *et al*. used at 14% cut-off point to distinguish the estrogen receptor-positive subtypes of breast cancer^53^. It was also reported by Aleskandarany *et al*. that 10% cut-off point was found to be important to distinguish low from highly proliferative tumors^54^. As for NETs, Rindi *et al*. proposed the grading as follows; grade 1 Ki67-positive rate <2%; grade 2 Ki67-positive rate between 3 and 20%; grade 3 Ki67-positive rate >20%^55^. Considering this reported evidence for the Ki67-positive rate, our result is supposed to be relatively lower than the cut-off points in cancer diagnosis, which corresponds to our finding of no tumorigenic transformation. In order to ensure safe cell transplantation, active proliferating cells and immature cells should be eliminated so as to avoid tumor formation. Additional induction culture for enhanced differentiation and cell surface markers purification might be considered for safer clinical application.

In conclusion, we report the motor functional recovery of murine sciatic nerves with a large lesion, by transplantation of LT-NCLCs derived from hiPSCs. LT-NCLC transplantation showed practical advantages, such as *in vivo* survival, axonal regrowth, remyelination, and angiogenesis inside the nerve conduits. LT-NCLCs from hiPSCs are promising candidate cells for clinical treatment of massive peripheral nerve defects.

## Electronic supplementary material

Supplementary dataset
