# Supplementary material for: Stem cells purified from human induced pluripotent stem cell-derived neural crest-like cells promote peripheral nerve regeneration
Source: Sci Rep. 2018 Jul 3;8:10071. doi: 10.1038/s41598-018-27952-7 (PMC6030210; doi:10.1038/s41598-018-27952-7)
Supplement: Supplementary file 1 — Supplementary dataset [file 41598_2018_27952_MOESM1_ESM.pdf]

Stem cells purified from human induced pluripotent stem cell-derived neural crest-like cells promote peripheral nerve regeneration

Hiroo Kimura, Takehito Ouchi, Shinsuke Shibata, Tsuyoshi Amemiya, Narihito Nagoshi, Taneaki Nakagawa, Morio Matsumoto, Hideyuki Okano, Masaya Nakamura, Kazuki Sato

Supplement Figure Legends, Supplement Table. 1, and Supplement Figure. 1 ~ 10

Supplement Table 1. List of antibodies used in this study.

Supplement Figure 1. LNGFR(+)THY-1(+) cells in NCLCs derived from 201B7, another iPSCs line.

Flow cytometric analysis was performed by using another iPSC line 201B7, differentiated into NCLCs. The induction efficiency for LNGFR(+)THY-1(+) was 20.5 ± 0.8%.

Supplement Figure 2. Flow cytometric analysis of single-positive and double-negative fraction.

Flow cytometric analysis revealed that single-positive [LNGFR(+)THY-1(-), LNGFR(-)THY-1(+)], and double-negative [LNGFR(-)THY-1(-) cells] fractions showed limited expression of NCC/MSC markers, including CD49d, CD57, CD29, CD73, CD105, CD140a and CD146.

Supplement Figure 3. NCC/MSC marker double-positive cells following NCLC induction.

Flow cytometric analysis revealed subsets of cells in the fraction double-positive for NCC/MSC markers, including NCC markers against LNGFR (CD271), CD49d and MSC markers against CD29, CD73, CD105, CD140a, CD146 and THY-1 (CD90).

Supplement Figure 4. Differentiated LT-NCLCs express both NCC and MSC markers.

Immunostaining of LT-NCLCs in NCC induction medium revealed that some differentiated LT-NCLCs were double-positive for the NCC marker CD57 and the MSC marker CD140a (arrow heads). Scale bar, 100 µm.

Supplement Figure 5. Expression level of NCC and MSC transcriptional factors in differentiated LT-NCLCs

(a) Various kinds of core transcriptional factors related to NCC / MSC development and differentiation were evaluated by immunocytochemistry in differentiated LT-NCLCs. The expression of FoxD3, Slug and AP2α were frequently detected–these are important for NCC lineage differentiation. High to intermediate levels of SOX9, Pax3, and Pax7 expression were detected; these are known as regulators of both NCC and MSC lineages. Among MSC lineage differentiation transcription factors, Foxp1 and GATA6 were moderately expressed, while Runx2 and PPARγ were

not expressed in differentiated LT-NCLCs. Scale bar, 50 µm.

(b) Quantitative data for the expression of these core transcriptional regulators are shown, indicating that differentiated LT-NCLCs exhibit heterogeneity for NCC and MSC lineages.

Supplement Figure 6. Surgical images of experimental groups.

(a-c) The exposed sciatic nerve was resected and repaired by nerve conduit.

(d-f) Re-exposed sciatic nerves at 12 weeks after the transplantation in the NC (d), TP (e), and Auto groups (f).

Supplement Figure 7. Migration of the grafted cells.

(a) Engrafted cells were stained with HNA and Hoechst. (b) Grafted cells migrated and evenly distributed throughout the nerve conduit in silicone tube. Scale bar, 100 µm. NS, non-significant.

Supplement Figure 8. Immunostaining of myelinated axons in control groups.

Images of immunohistochemistry of the NC, Auto, and Sham groups, stained for P0, Peripherin, and STEM12 in middle axial sections at 12 weeks after transplantation. Myelinated axons were slightly detected in the NC group. No STEM121-positive cells were detected in all groups. Scale bar, 10 µm.

Supplement Figure 9. Disappearance of the NCC/MSC markers in grafted cells.

Immunohistochemical analysis for the engrafted cells stained with NCC marker CD49d and MSC marker CD146 with Hoechst demonstrated that survived engrafted cells lost expression of NCC/MSC markers 12-weeks after transplantation. Scale bar, 100 µm.

Supplement Figure 10. Expression of neurotrophic and angiogenic factors in differentiated LT-NCLCs.

Differentiated LT-NCLCs started to express neurotrophic factor BDNF and angiogenic factor VEGF more strongly compared to iPSCs. Scale bar, 50 µm.

Marker List (FACS; fluorescence activated cell sorting, IHC; immunohistochemistry, ICC; immunocytochemistry, iEM; immuno-electron microscopic analysis)

**Primary Antibody**	**Property**	**Company**	**Host**	**Clone**	**Use(conjugation/dilution)**
CD271, low-affinity nerve growth factor receptor; LNGFR	Mesenchymal stem cell marker, Neural crest lineage cell marker	Miltenyi Biotec, Bergisch Gladbach, Germany	mouse	ME20.4-1.H4	FACS (APC)
CD90, thymocyte antigen-1; THY-1	Mesenchymal stem cell marker	BD Biosciences, CA, USA	mouse	5E 10	FACS (FITC)
CD49d, integrin alpha-4	Neural crest lineage cell marker	BioLegend, CA, USA	mouse	9F10	FACS (PE Cy7)<br>IHC (1:50)
CD57, human natural killer-1; HNK-1	Neural crest lineage cell marker	Chemicon, Darmstadt, Germany	mouse	NK-1	FACS (PE)<br>ICC (1:100)
CD29, integrin beta-1	Mesenchymal stem cell marker	BioLegend, CA, USA	mouse	TS2/16	FACS (PE)
CD73, 5'-nucleotidase; 5-NT	Mesenchymal stem cell marker	BioLegend, CA, USA	mouse	AD2	FACS (PE)
CD105, endoglin	Mesenchymal stem cell marker	BioLegend, CA, USA	mouse	43A3	FACS (PE)
CD140a, platelet-derived growth factor receptor alpha; PDGFRα	Mesenchymal stem cell marker	BioLegend, CA, USA	mouse	16A1	FACS (PE)<br>ICC (1:100)
CD146, melanoma cell adhesion molecule; MCAM	Mesenchymal stem cell marker	BioLegend, CA, USA	mouse	SHM-57	FACS (PE)<br>IHC (1:50)
FoxD3, forkhead box protein D3	Neural crest lineage cell marker	Abcam, Cambridge, UK	rabbit	<polyclonal>	ICC (1:250)
Slug, Snail2, Snail homolog 2	Neural crest lineage cell marker	Abcam, Cambridge, UK	rabbit	<polyclonal>	ICC (1:500)
AP2α, activator protein 2 alpha	Neural crest lineage cell marker	Cell Signaling Technologies, MA, USA	rabbit	C83E10	ICC (1:100)
SOX9, SRY-related HMG-box 9	Mesenchymal stem cell marker, Neural crest lineage cell marker	Santa Cruz Biotechnology, TX, USA	rabbit	<polyclonal>	ICC (1:400)
Pax3, paired box gene 3	Mesenchymal stem cell marker, Neural crest lineage cell marker	Abcam, Cambridge, UK	goat	<polyclonal>	ICC (1:200)
Pax7, paired box gene 7	Mesenchymal stem cell marker, Neural crest lineage cell marker	Santa Cruz Biotechnology, TX, USA	mouse	PAX7	ICC (1:200)
FoxP1, forkhead box protein P1	Mesenchymal stem cell marker	OriGene Technologies, Inc., MD, USA	mouse	UMAB89	ICC (1:100)
GATA6, GATA-binding factor 6	Mesenchymal stem cell marker	Thermo Fisher Scientific, MA, USA	rabbit	<polyclonal>	ICC (1:200)
Runx2, Runt-related transcription factor 2	Mesenchymal lineage cell marker	Abcam, Cambridge, UK	mouse	Runx2	ICC (1:100)
PPARγ, Peroxisome proliferator-activated receptor gamma	Mesenchymal lineage cell marker	Santa Cruz Biotechnology, TX, USA	mouse	E-8	ICC (1:200)
SOX10, SRY-related HMG-box 10	Neural crest lineage cell marker	R&D Systems, MN, USA	goat	<polyclonal>	ICC (1:200)
Peripherin	Neural cell marker	Chemicon, Darmstadt, Germany	rabbit	<polyclonal>	ICC (1:200)<br>IHC (1:200)
Krox20, Early growth response protein 2; EGR2	Schwann cell lineage marker	Covance, NJ, USA	rabbit	<polyclonal>	ICC (1:100)
OCT6, Octamer-binding factor 6 (Oct-6/Pou3f1)	Schwann cell lineage marker	Santa Cruz Biotechnology, TX, USA	goat	<polyclonal>	ICC (1:200)
MBP, myelin basic protein	Schwann cell lineage marker	Abcam, Cambridge, UK	rat	12	ICC (1:200)
P0, protein myelin zero	Schwann cell lineage marker	Aves Labs, OR, USA	chicken	<polyclonal>	ICC (1:200)<br>IHC (1:200)
HNA, Human nuclear antigen; HNA	Human cell specific marker	Chemicon, Darmstadt, Germany	mouse	235-1	IHC (1:200)
Ki67	Cell proliferation marker	Novocastra, Newcastle, UK	rabbit	<polyclonal>	IHC (1:1000)

Human Cytoplasmic Marker, STEM121	Human cell specific marker	Stem Cells, Inc., CA, USA	mouse	AB-121-U-050	IHC (1:1000), iEM (1:500)
mouse CD31, platelet endothelial cell adhesion molecule-1; PECAM-1	Endothelial cell marker	BD Biosciences, CA, USA	rat	MEC13.3	IHC (1:50)
human CD31, platelet endothelial cell adhesion molecule-1; PECAM-1	Endothelial cell marker	BD Biosciences, CA, USA	mouse	WM59	IHC (1:100)
BDNF, Brain-derived neurotrophic factor	Signaling proteins (neurotrophic)	Abcam, Cambridge, UK	rabbit	<polyclonal>	ICC (1:250)
VEGF, Vascular endothelial growth factor	Signaling proteins (angiogenic)	Bioss, MA, USA	rabbit	<polyclonal>	ICC (1:250)

**Secondary Antibody**	**Property**	**Company**	**Host**	**Clone**	**Use(conjugation/dilution)**
Goat anti-mouse/rabbit/chicken/rat IgG, anti-mouse IgM	species specific IgG detection	Thermo Fisher Scientific, MA, USA	goat	<polyclonal>	ICC/IHC (1:1000) Alexa488/555/647
Donkey anti-mouse/rabbit/goat IgG	species specific IgG detection	Thermo Fisher Scientific, MA, USA	donkey	<polyclonal>	ICC/IHC (1:1000) Alexa488/555/647

Supplementary Table 1. List of antibodies used in this study

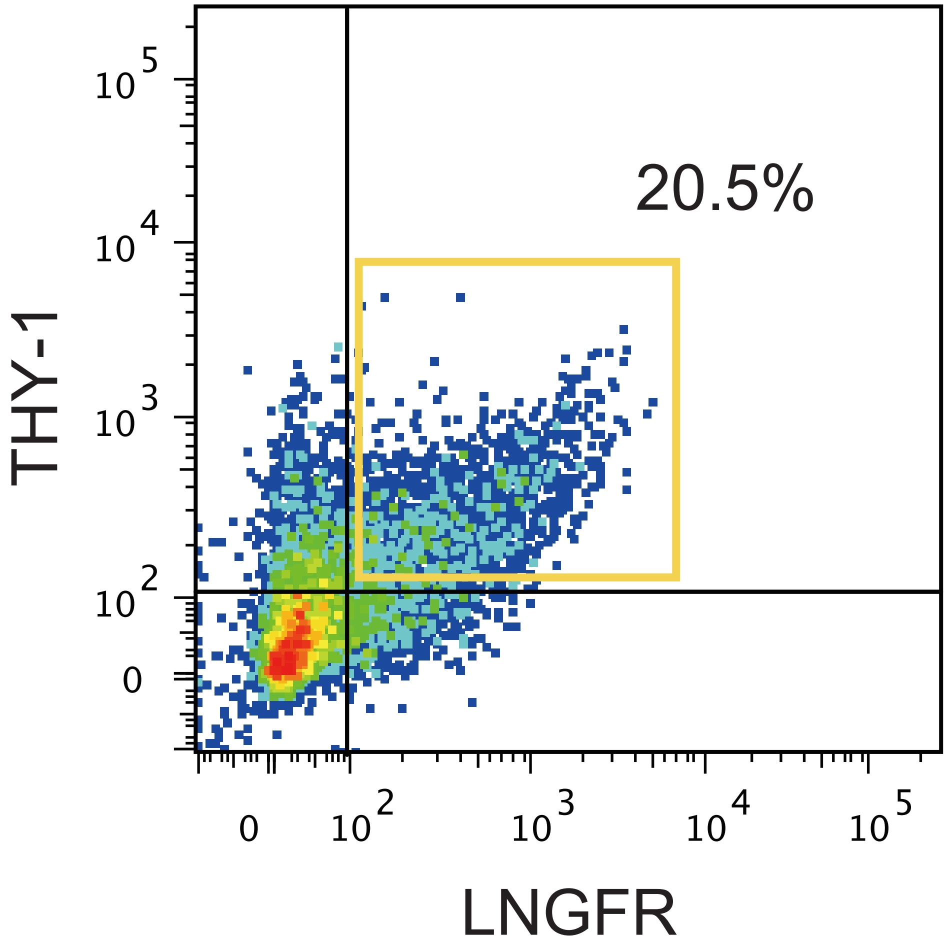

Supplement Figure. 1

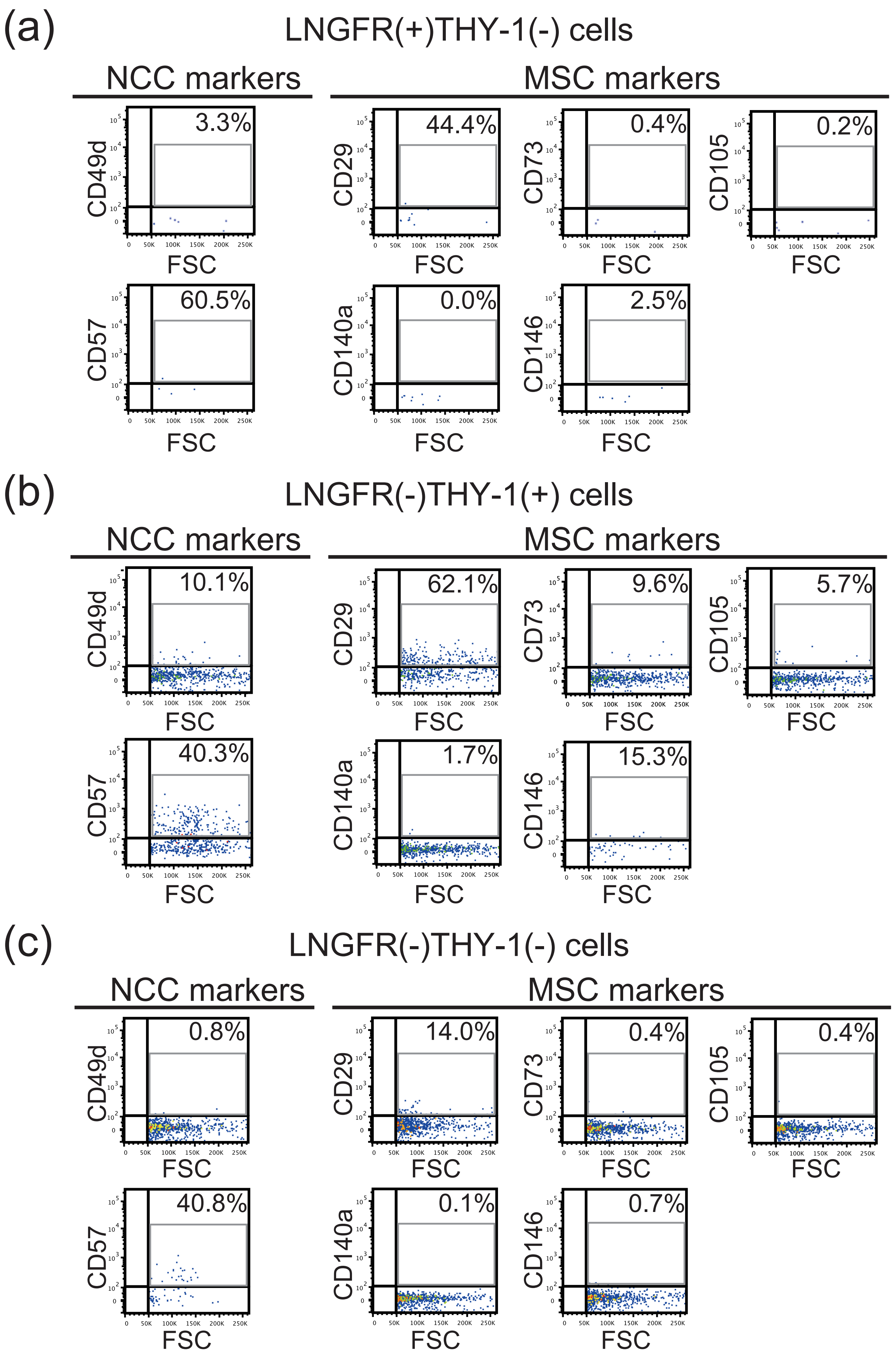

Supplement Figure. 2

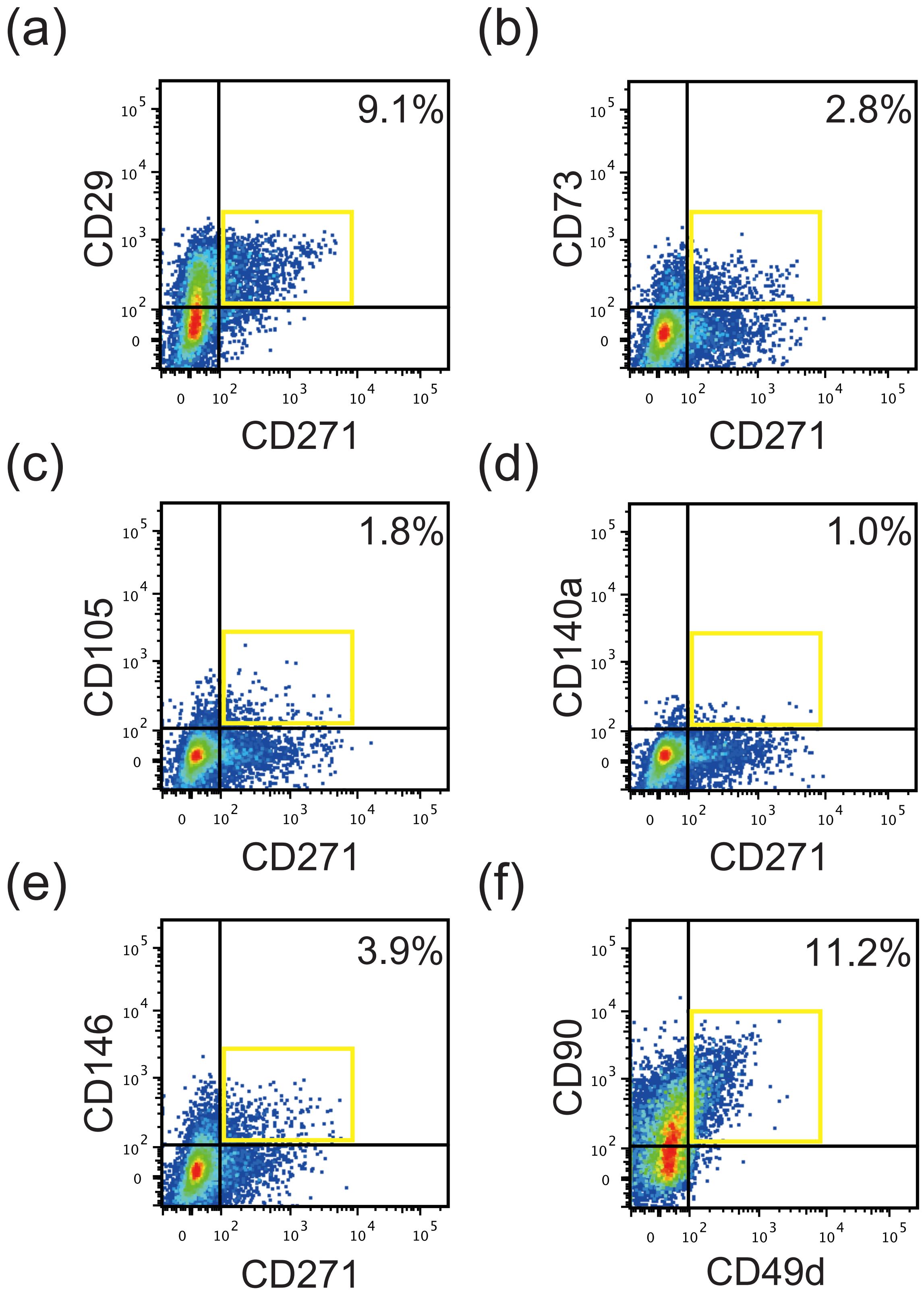

Supplement Figure. 3

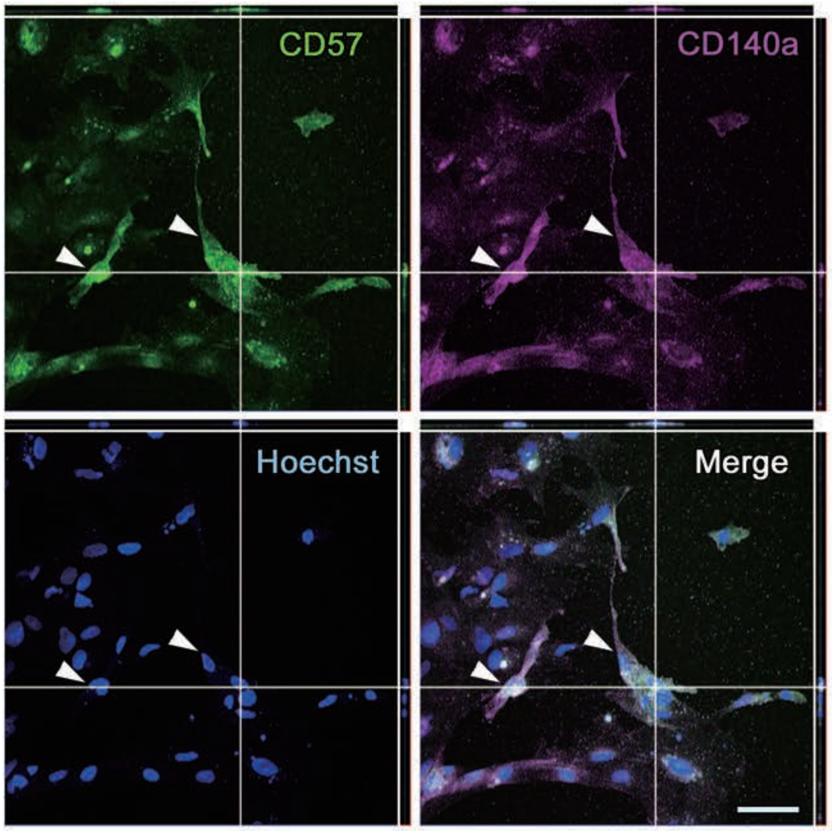

Supplement Figure. 4

(a)

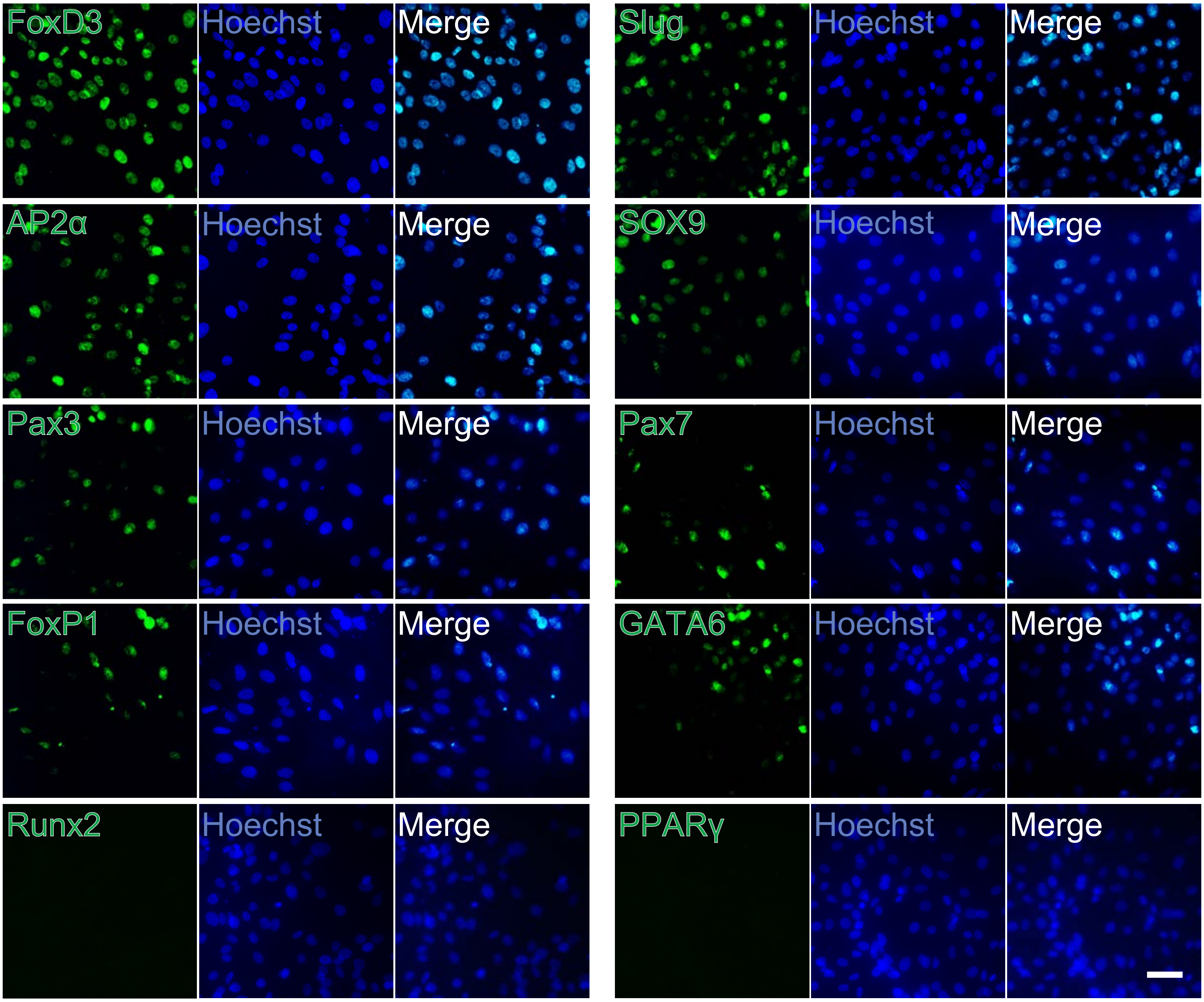

(b)

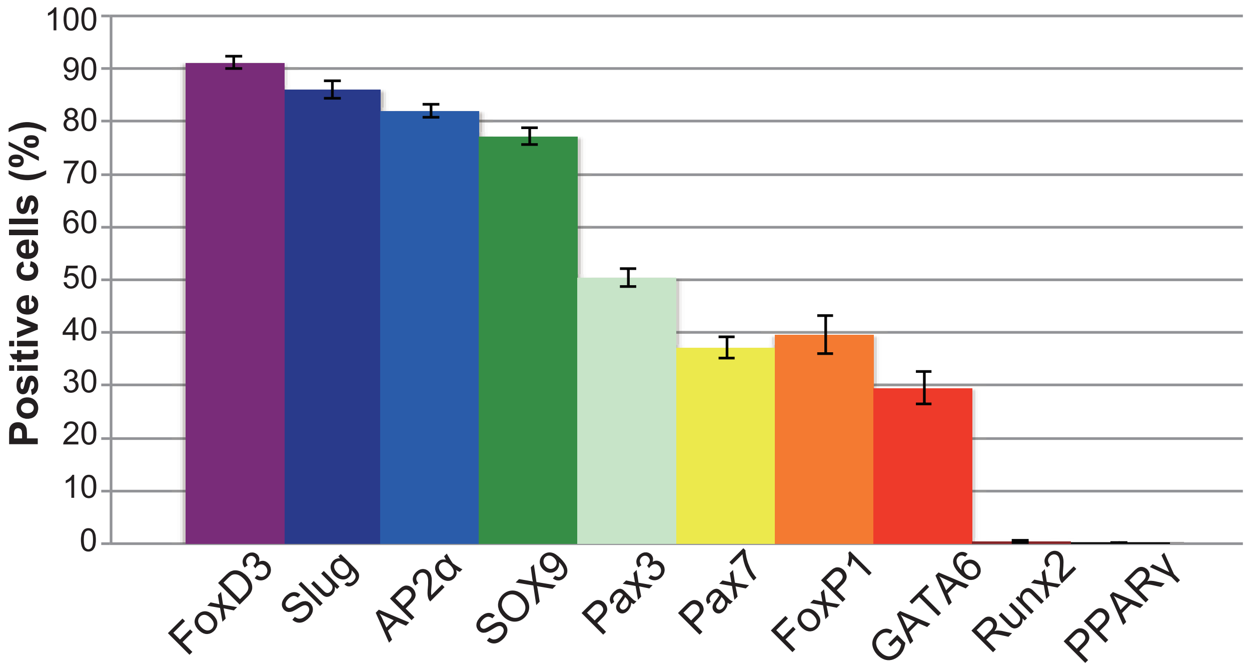

Supplement Figure. 5

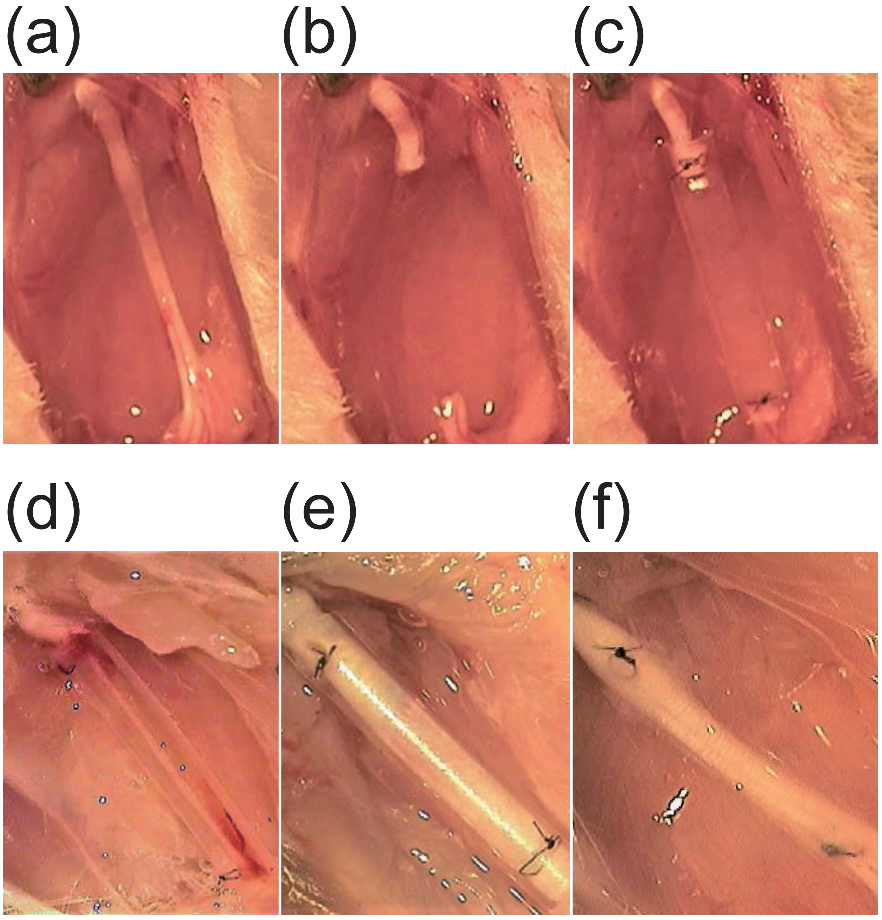

Supplement Figure. 6

(a)

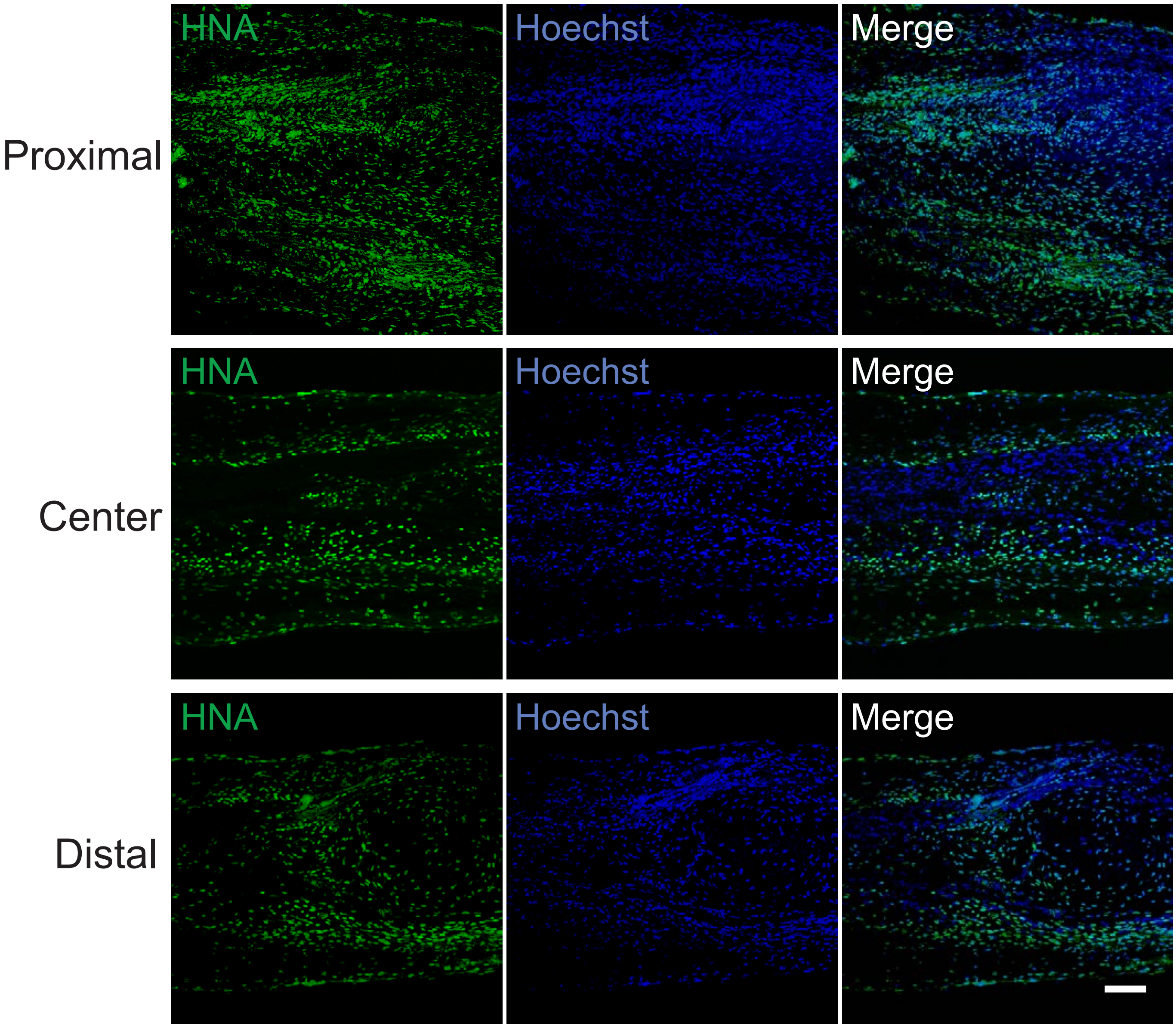

(b)

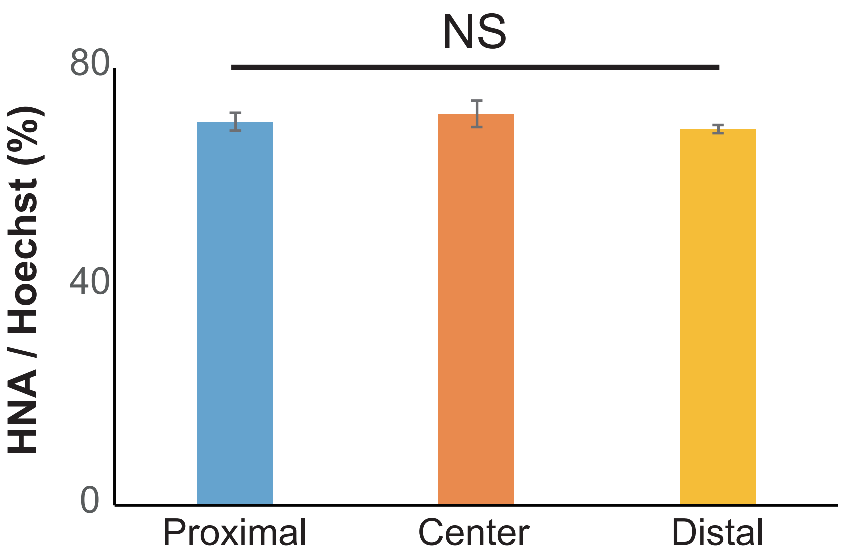

Supplement Figure. 7

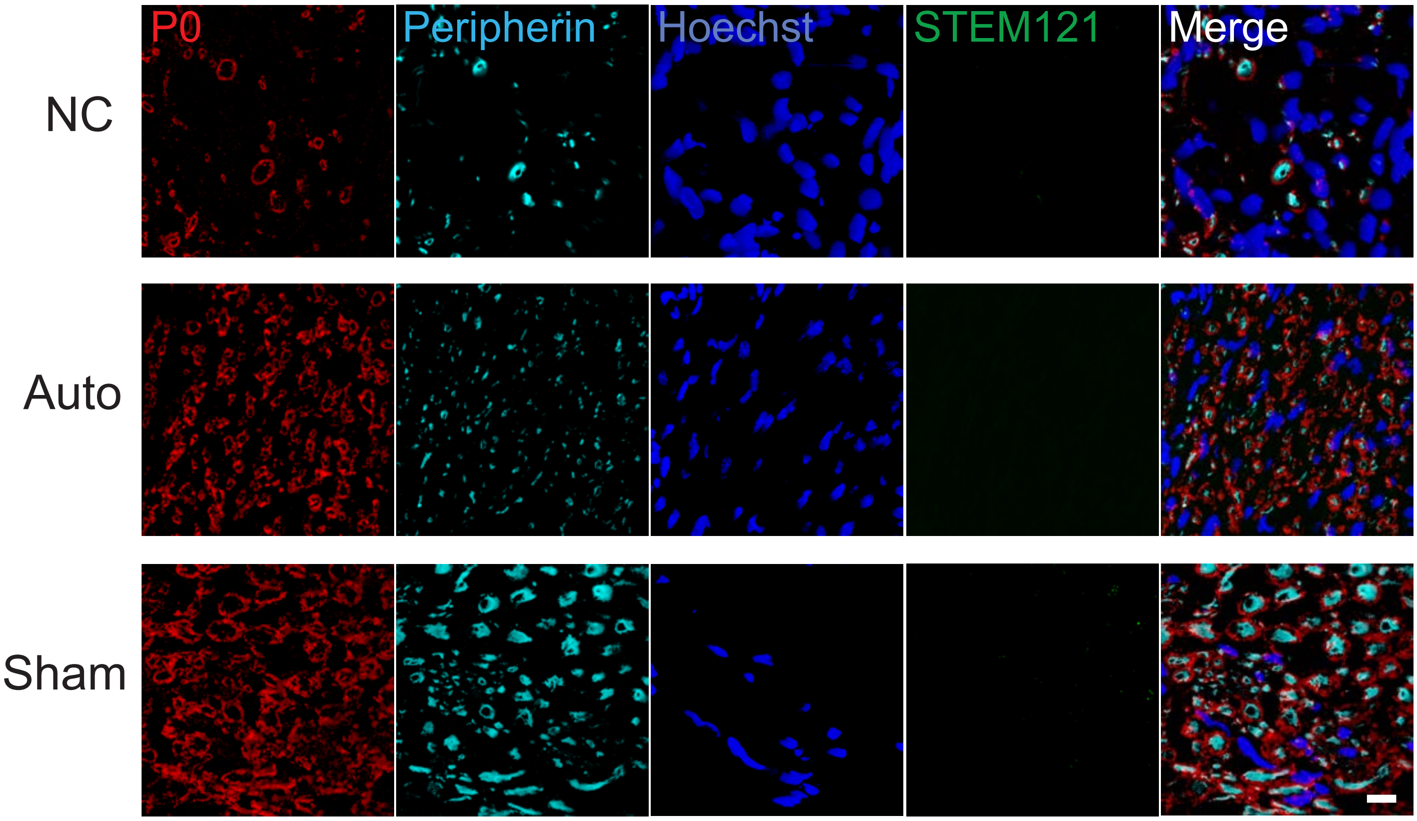

Supplement Figure. 8

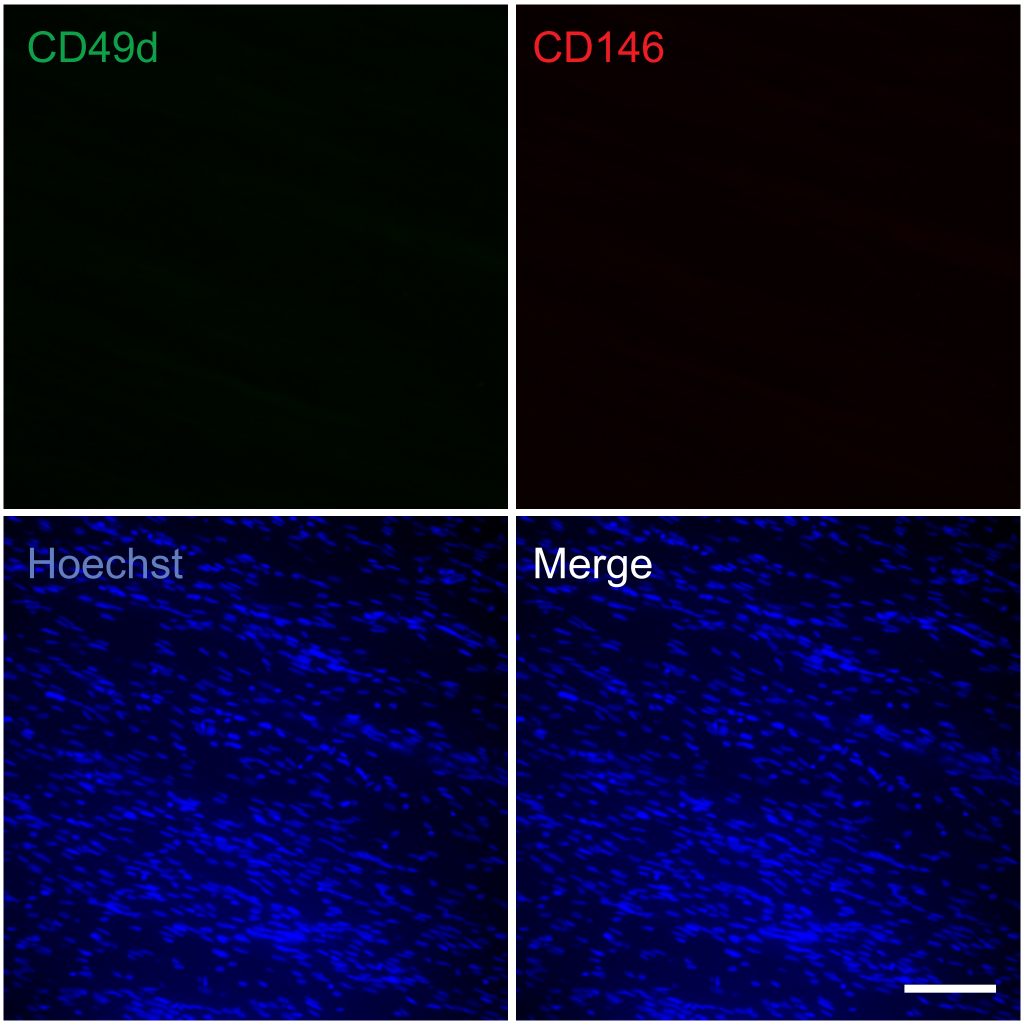

Supplement Figure. 9

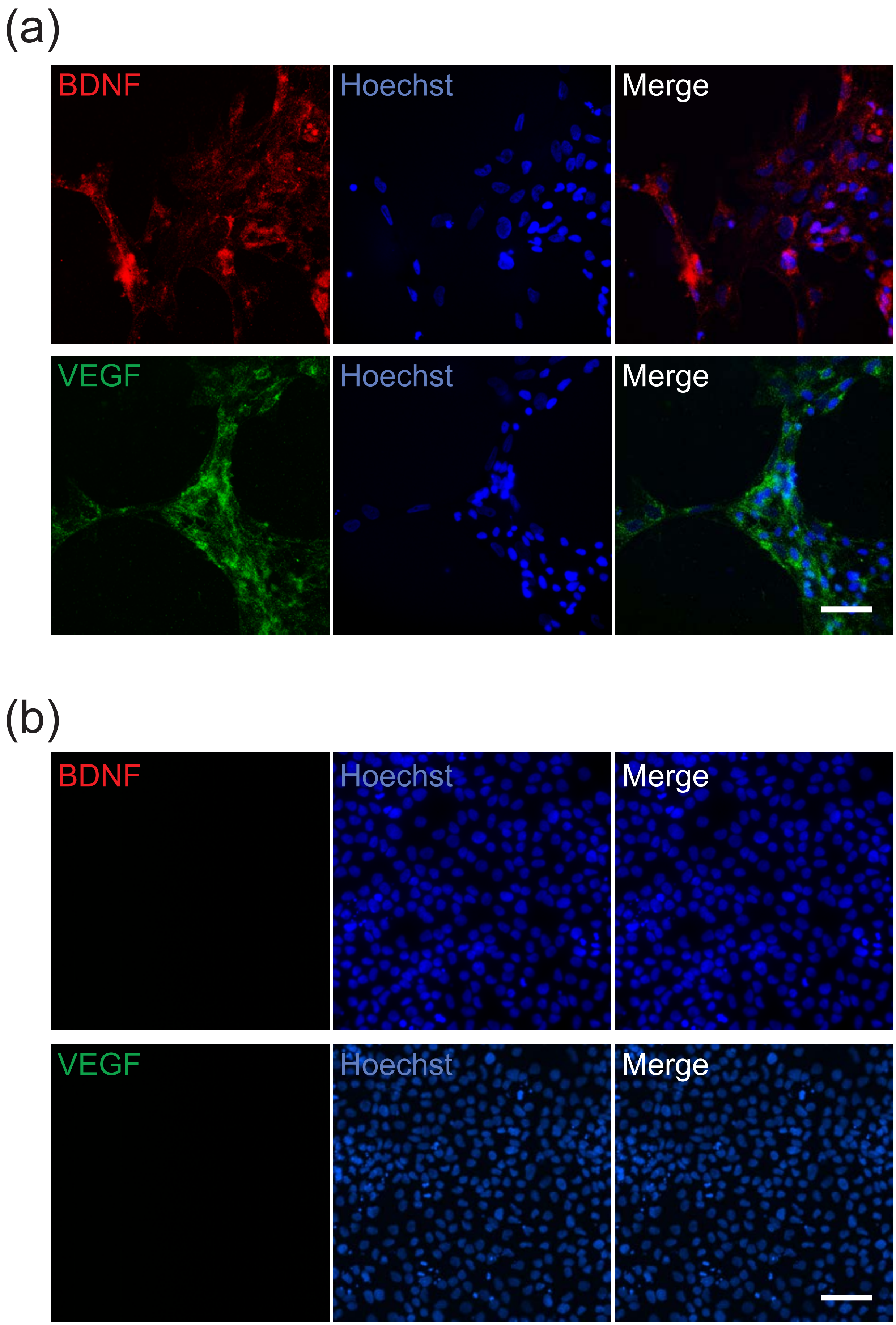

Supplement Figure. 10
